# Emotional Mental Imagery as Simulation of Reality: Fear and Beyond—A Tribute to Peter Lang

**DOI:** 10.1016/j.beth.2015.11.004

**Published:** 2016-09

**Authors:** Julie L. Ji, Stephanie Burnett Heyes, Colin MacLeod, Emily A. Holmes

**Affiliations:** Medical Research Council Cognition and Brain Sciences Unit, Cambridge; University of Oxford; University of Birmingham; University of Western Australia; Babeş-Bolyai University, Romania; Medical Research Council Cognition and Brain Sciences Unit, Cambridge; Karolinska Institutet, Stockholm

**Keywords:** mental imagery, visual imagery, emotion, cognitive therapy, behavior therapy

## Abstract

This article pays tribute to the seminal paper by Peter J. Lang (1977; *this journal*), “Imagery in Therapy: Information Processing Analysis of Fear.” We review research and clinical practice developments in the past five decades with reference to key insights from Lang’s theory and experimental work on emotional mental imagery. First, we summarize and recontextualize Lang’s bio-informational theory of emotional mental imagery (1977, 1979) within contemporary theoretical developments on the function of mental imagery. Second, Lang’s proposal that mental imagery can evoke emotional responses is evaluated by reviewing empirical evidence that mental imagery has a powerful impact on negative as well as positive emotions at neurophysiological and subjective levels. Third, we review contemporary cognitive and behavioral therapeutic practices that use mental imagery, and consider points of extension and departure from Lang’s original investigation of mental imagery in fear-extinction behavior change. Fourth, Lang’s experimental work on emotional imagery is revisited in light of contemporary research on emotional psychopathology-linked individual differences in mental imagery. Finally, key insights from Lang’s experiments on training emotional response during imagery are discussed in relation to how specific techniques may be harnessed to enhance adaptive emotional mental imagery training in future research.

Scientific interest in mental imagery dates back to the 19th century (Galton, 1880), making imagery one of the most enduring topics in psychological science. Despite such extended interest in the phenomenon of imagery, Peter Lang was one of the first scientists to formulate a testable theory of emotion-inducing mental imagery. Lang, 1977, Lang, 1979 of emotional imagery was originally developed to explain the role of mental imagery in facilitating behavioral fear extinction in imaginal exposure therapy. Lang, 1977, Lang, 1979 theory opened an experimental window onto “the mind’s emotional eye,” and has spurred research and influenced clinical practice in the ensuing decades. In reviewing theoretical, empirical, and clinical developments related to emotional mental imagery, we aim to highlight the contemporary relevance of Lang’s theory, while drawing attention to key insights that we believe are capable of exerting a beneficial impact on future research and clinical practice in years to come.

### Lang’s Bio-informational Theory of Emotional Imagery

Mental imagery refers to perceptual experience in the absence of sensory input, commonly described as seeing with the “mind’s eye,” hearing with the “mind’s ear,” and so on (Kosslyn, Ganis, & Thompson, 2001). In this paper we consider mental imagery both as an emotion-evoking stimulus that can be manipulated (e.g., during therapeutic techniques such as imaginal exposure; Foa, Hembree, & Rothbaum, 2007), and as a symptom of psychopathology (e.g., distressing intrusive memories/flashbacks in posttraumatic stress disorder; cf. Holmes & Mathews, 2010).

In his bio-informational theory of emotional imagery, Lang, 1977, Lang, 1979 postulated that a mental imagery representation of an emotionally charged stimulus (e.g., a spider) activates an associative network of stored information that overlaps with that activated during actual experience of the stimulus in reality (e.g., encountering a live spider).

This associative network of information is said to consist of perceptual information about the stimulus (color, shape, size, texture of spider), semantic information about what it means (insect, danger, bite), somatovisceral response information about what it feels like to encounter the stimulus (fear, racing heart), and preparatory motor responses evoked by the encounter (e.g., muscles tensing to flee from the spider). According to this theory, mental imagery differs from verbal thought in that only mental imagery has the capacity to activate physiological and behavioral response systems (Lang, 1987). Research pertaining to this assertion will be discussed in Section III.

Lang, 1977, Lang, 1979 associative-network information processing definition of mental imagery was influenced by Pylyshyn's (1973) propositional theory of mental imagery, which construed mental imagery as conceptual representations describing reality, rather than as pictorial representations depicting reality. Historically, the debate concerning whether mental imagery involves conceptual representations (Pylyshyn, 1973) or pictorial representations (Kosslyn, 1981) has been the subject of heated debate. Neuroimaging and psychophysics evidence gathered in the past 50 years has largely resolved the debate in favor of the latter view (see Pearson, Naselaris, Holmes, & Kosslyn, 2015, for a review). Although this appears to refute the grounds of Lang’s bio-informational theory, closer inspection reveals that the validity of this theory stands irrespective of whether mental imagery is conceptual or pictorial in nature.

As Lang (1987) explicated, the bio-informational theory was developed not as a theory on the nature of mental imagery, but as a functional theory concerning the impact of mental imagery on emotional processing. Due to the overlap in perceptual information between imagined and real stimuli, Lang, 1977, Lang, 1979 proposed that imagined interaction with a stimulus can evoke corresponding emotional responses associated with real interaction with that stimulus. As such, imagined interaction with stimuli can function as an “as-if real” template for rehearsing and modifying emotional and behavioral responses to the same stimuli in real life. Lang, 1977, Lang, 1979 proposed that this function of mental imagery could be harnessed in clinical treatment to facilitate fear-extinction learning and habituation via the rehearsal and learning of new adaptive responses during imaginal exposure therapy.

Indeed, numerous studies using a range of associative learning paradigms have shown that mental imagery can produce conditioned responses in the same way as real stimuli (cf. Dadds et al., 1997, Lewis et al., 2013). Crucially, Lang proposed that the elicitation of a fear emotional response during mental imagery of the feared stimuli (simulation of both perceptual representations and autonomic and behavioral responses) is required for learning to occur, and is therefore necessary in order for imaginal exposure to be effective (Lang, 1977, Wolpe, 1958). Interestingly, Lang’s conception of mental imagery as an “as-if real” template parallels contemporary functional perspectives on mental imagery. These contemporary accounts view mental imagery as a core component of the "prospective brain," which enables the simulation of hypothetical future events based on prior knowledge and memories of past experience for the purposes of prediction and planning (Moulton and Kosslyn, 2009, Schacter et al., 2008, Suddendorf and Corballis, 2007).

Of particular relevance to Lang, 1977, Lang, 1979 functional theory is Moulton and Kosslyn's (2009) theory of mental imagery as emulation. Emulation is defined as the episodic construction of a hypothetical scenario that simulates not only perceptual information about an event, but also rich semantic and affective information about plausible causes and consequences of the imagined scenarios (Moulton & Kosslyn, 2009). As such, both Lang, 1977, Lang, 1979 and Moulton and Kosslyn (2009) postulate that mental imagery has the capacity to evoke cognitive and emotional responses, enabling the individual to not only “try out” one or more versions of what might happen (Schacter et al., 2008, p. 40), but to also “try out” the emotional consequences of alternative courses of action (Lang, 1987, p. 412; Moulton & Kosslyn, 2009, p. 1278).

By providing a pivotal testable framework linking mental imagery to emotion, Lang, 1977, Lang, 1979 bio-informational theory paved the way for much subsequent research concerning the functions of mental imagery. While Lang focused on the role of imagery in fear extinction learning, contemporary theorists point to the wider role of mental imagery in planning, problem solving, and self-regulation (Gilbert and Wilson, 2007, Suddendorf and Corballis, 2007, Taylor et al., 1998).

The first half of this review will evaluate evidence pertaining to Lang’s key proposal, that mental imagery has the capacity to evoke emotional responses, and explore how this capacity has been exploited therapeutically in clinical treatment. The second half of the review will consider how subsequent research has built on Lang’s initial work concerning individual differences to illuminate how variability in imagery may relate to emotional dysfunction, and highlight neglected insights from Lang’s research on emotional imagery training that could potentially contribute to future treatment innovation.

## Section I: Mental Imagery-Evoked Emotional Response

Developments in clinical psychology over the past 50 years have shown that unwanted and distressing mental imagery is a symptom present in a wide range of anxiety and mood disorders (Holmes & Mathews, 2010). In anxiety disorders, mental imagery phenomena include the intrusive flashbacks that define posttraumatic stress disorder (Brewin and Holmes, 2003, Ehlers et al., 2002), and imagery of feared stimuli or distorted images of one’s own physical appearance in the case of phobias or social phobia, respectively (Hirsch & Holmes, 2007). Depression and bipolar disorder symptomology also feature mental imagery of past failures and trauma (Kuyken & Brewin, 1994), as well as possible future events, such as suicidal acts (Holmes, Crane, Fennell, & Williams, 2007).

While clinical presentation suggests that negative mental imagery symptomology can have emotionally distressing consequences for patients, researchers have noted a relative paucity of studies designed to directly evaluate this assumption (Holmes and Mathews, 2010, Watts, 1997). Lang, 1977, Lang, 1979 was one of the first investigators to empirically examine the capacity for mental imagery to evoke emotional response. Much of Lang’s experimental work on mental imagery was devoted to assessing psychophysiological reactivity to mental imagery cued by verbal scripts depicting emotional scenarios and bodily responses (e.g., “your face is flushed as your muscles strain to continue the pace”). In this section, we review evidence resulting from the work of Lang, and others, that mental imagery does indeed have an impact on emotion, as indexed by physiological, neurological, and subjective measures. Furthermore, response information measured at these levels has also revealed biases in emotional processing in individuals suffering from emotional psychopathology, such as mood and anxiety disorders.

### The Impact of Emotional Mental Imagery on Physiological Activity

It is widely accepted that emotional experience involves activation of the central and peripheral nervous systems (Gross & Barrett, 2011). Thus, if mental imagery representations of emotional scenarios are capable of eliciting emotional responses in an “as-if real” manner (Kosslyn et al., 2001, Kreiman et al., 2000, Lang, 1979), this may be observable at a physiological level.

Using verbal scripts depicting emotionally negative or neutral information, Lang, 1977, Lang, 1979 experimental work measured peripheral nervous system responses while participants imagined the content of the scripts. The central hypothesis was that physiological effects would arise during emotional imagery as a result of activation of perceptual memories, triggering somatovisceral responses associated with experience of the stimuli in reality.

In terms of autonomic arousal, Lang and colleagues observed greater heart rate acceleration during mental imagery of high versus low arousal scenarios. In healthy participants, imagery of fear and anger-related scenarios led to greater heart rate acceleration than did imagery of neutral scenarios (Cook et al., 1991, Vrana, 1995, Vrana et al., 1986, Witvliet and Vrana, 1995). Similarly, skin conductance response (SCR) levels also indicated elevated emotional arousal during mental imagery of emotional scenarios, such that scripts depicting highly arousing pleasant and unpleasant experiences produced larger increases in SCR relative to those depicting neutral experiences, in healthy populations (Lang et al., 1983, Weerts and Lang, 1978).

Research has shown that respiratory responses are also influenced by mental imagery in healthy populations. Specifically, imagery evoked by scripts depicting high arousal scenes (fear and action-related) compared to low arousal scenes (relaxation and depression-related) produced greater drops in end-tidal fractional carbon dioxide concentration, likely reflecting hyperventilation (Van Diest et al., 2001). Importantly, this hyperventilation during emotional imagery was more pronounced in individuals with higher relative to lower imagery generation ability, as assessed using the Questionnaire Upon Mental Imagery (QMI; Sheehan, 1967). This individual differences dimension in imagery has also been explored in Lang’s work, and will be considered in more detail in Section III.

In addition to autonomic indices, mental imagery has been shown to modulate other indices of physiological arousal, such as the startle blink reflex. Vrana and Lang (1990) required healthy participants to first learn and then recall six pairs of sentences depicting fear-related and neutral scenarios. During recall, participants were instructed either to relax and ignore the sentence (control condition), to silently articulate the sentence (verbal condition), or to imagine the sentence content as a personal experience (imagery condition). As expected, startle blink reflexes evoked by acoustic probes were found to be greater during recall of fear relative to neutral sentences. Importantly, this effect was greater in the imagery condition than in either the control condition or the verbal condition (Cuthbert et al., 2003).

Finally, mental imagery of food stimuli has been shown to modulate the gustatory salivary reflex. Research in the field of brain computer interfaces (BCI) has found increases and decreases in salivary pH levels compared to baseline as a result of a healthy participant imagining consuming a lemon versus drinking milk, respectively (Vanhaudenhuyse, Bruno, Bredart, Plenevaux, & Laureys, 2007). This finding has also been harnessed to demonstrate conscious awareness in clinical patients with complete locked-in syndrome (LIS), individuals who cannot otherwise indicate the presence of conscious awareness (Wilhelm, Jordan, & Birbaumer, 2006). In addition to appetitive salivary responses, repetitive mental imagery of food consumption (eating M&Ms) has also been shown to lead to food item-specific satiation effects (Morewedge, Huh, & Vosgerau, 2010). Although no physiological measures were included in the study, consumption of that food item was reduced in the high-repetition imagery group (30 repetitions) relative to the low-repetition imagery group (three repetitions), indicating satiation effects (Morewedge et al., 2010).

Together, the above evidence provides support for Lang, 1977, Lang, 1979 contention that mental imagery has the capacity to activate the peripheral nervous system and evoke somatic responses, be it in the form of “fight or flight” sympathetic system response during fear- or anger-related imagery, or “rest and digest” parasympathetic response during gustatory imagery. However, one limitation associated with using physiological indicators of autonomic nervous system (ANS) activation is that these can be employed only to index high arousal emotions, such as anger, fear, disgust, or elation. In contrast, both high and low arousal emotional responses can be assessed using neurological measures, such as those provided by neuroimaging.

Furthermore, the studies reviewed in this section have not explicitly contrasted mental imagery representations of emotional stimuli to an alternative mode of representation of the same information, and therefore one cannot conclude based on the evidence that the physiological effects observed reflect the impact of mental imagery *per se*, rather than the impact of emotional information processing in general. Research addressing this limitation will be discussed in Section III.

### The Impact of Emotional Mental Imagery on Neural Measures of Emotional Response

According to Lang's (1979) bio-informational theory, emotional imagery is hypothesized to be able to elicit similar physiological responses in both peripheral and central systems as would be evoked during actual experience (Lang & McTeague, 2009). Since the 1970s, developments in brain imaging technology have enabled assessment of the impact of emotional imagery on central nervous system (CNS) structures involved in coordinating peripheral nervous system (PNS) responses.

Neural indices of emotion processing during emotional mental imagery can be evaluated in comparison to activity observed during veridical perception of emotional stimuli, or to emotionally neutral mental imagery. Meta-analytic reviews of neuroimaging studies on healthy participants show that the brain regions most consistently associated with emotional processing are the dorsomedial prefrontal cortex (mPFC), anterior cingulate cortex (ACC) and amygdala (Murphy et al., 2003, Phan et al., 2002), and the insular cortex (Craig, 2009), all of which are involved in the coordination of ANS activity. Activation of these same emotion-processing regions has been observed during emotional mental imagery. Early hemodynamic neuroimaging studies using positron emission tomography (PET) showed that mental imagery of emotional relative to neutral information elicited increased regional cerebral blood flow (rCBF) to the mPFC, ACC and anterior insula (Kosslyn et al., 1996, Partiot et al., 1995, Schaefer et al., 2003). This effect has been found in PET studies not only for mental imagery evoked by scripts depicting negative scenarios (aggression and guilt-related) (Shin et al., 2000), but also for mental imagery evoked by scripts depicting positive scenarios (success and affection-related) (Schaefer et al., 2003).

More recent studies have used script-driven emotional imagery to examine the specificity of neural circuits for the processing of emotional valence versus arousal. Costa, Lang, Sabatinelli, Versace, and Bradley (2010) used functional magnetic resonance imaging (fMRI) to examine negative, positive, and neutral script-driven imagery, and found that activation of the amygdala was enhanced during emotional relative to neutral imagery, irrespective of emotional valence. However, activation of the nucleus accumbens (NAc) and mPFC were selectively enhanced during positive relative to neutral imagery, not negative relative to neutral imagery (Costa et al., 2010). Results from this script-driven imagery study are also consistent with a previous fMRI study where participants viewed positive and neutral pictures (Sabatinelli, Bradley, Lang, Costa, & Versace, 2007).

Studies using fMRI have also compared neural responses to real versus imagined emotional stimuli in the same participants. Kim et al. (2007) found comparable magnitudes of left hemisphere amygdala activity between when participants viewed faces with negative and positive emotional expressions and when they generated mental imagery of such faces. In fMRI studies using real-time neural activation feedback (“neurofeedback”), over successive trials participants were able to use self-generated visualization of positive and negative scenarios to regulate the activation of emotion processing regions such as the insula (S. Lee et al., 2011). In addition to face stimuli, mental imagery of positive and negative events in the past and future has been shown to activate the amygdala and ACC (Sharot, Riccardi, Raio, & Phelps, 2007). In another study examining negative and positive mental imagery in the same participants, Damasio et al. (2000) found different ACC subregions were differentially activated by fear, anger, happiness, and sadness imagery.

Finally, direct evidence that visual imagery and visual perception share common neural systems implicated in emotional response comes from a single-cell recording study on human epileptic patients. Kreiman et al. (2000) found that 75% of the 89 recorded cells in the amygdala selectively altered their firing rates in comparable patterns during both visual perception and visual imagery recall of emotional faces, whereas 4% and 10% responded selectively to imagery or veridical perception, respectively (Kreiman et al., 2000).

As such, results from neuroimaging and single cell recording studies provide converging evidence that mental imagery has the capacity to evoke an emotional response by activating neural networks involved in emotional processing and response coordination with the autonomic nervous system. However, as with the physiology studies reviewed earlier, the neuroimaging studies reviewed here have not explicitly contrasted mental imagery representations of emotional stimuli to an alternative mode of representation of the same information, and therefore one cannot conclude based on the evidence that the physiological effects observed reflect the impact of mental imagery *per se*, rather than the impact of emotional information processing in general.

### Disruption of Emotional Mental Imagery Reduces Emotional Impact

If mental imagery-based representation of emotional information serves to elicit emotional responses to this information, then it follows that *disruption* of such imagery should reduce the intensity of such emotional responding. This hypothesis has been investigated using dual task paradigms that reduce the availability of cognitive resources required for mental imagery generation. Typically, in such studies, participants attempt to generate mental imagery while undertaking a concurrent task that consumes working memory resources, such as sequenced finger tapping on a number pad, or mental arithmetic.

Across several experiments, Andrade, Kavanagh, and Baddeley (1997) asked healthy participants to generate visual imagery from emotionally negative versus neutral cues (photographs and personal memories) while concurrently tapping a spatial pattern with a finger, engaging in lateral eye movements, or performing no concurrent task. Results showed lower vividness of mental imagery in the concurrent spatial tapping and lateral eye movement conditions, relative to the no-concurrent task control condition. Critically, this reduction in the imagery vividness was accompanied by a corresponding decline in the intensity of participants’ emotional responding to the negative, compared to neutral, cues. This result was replicated by Kavanagh, Freese, Andrade, and May (2001) using a within-subjects design in which participants generated both positive and negative autobiographical episodic memory imagery at three time points. At the first and third time points, imagery generation took place without a concurrent imagery interference task, whereas at the second time point, imagery interference was produced by concurrent performance of a lateral eye-movement task and viewing of dynamic visual noise, in counterbalanced order. Concurrent imagery interference was successful in reducing the vividness ratings for negative (but not positive) memories, as compared to the no-interference condition. Again, the critically important finding was that performance of the concurrent task also served to dampen the emotional impact of these memories.

Clinical researchers have also begun to harness the potential for concurrent tasks to reduce the emotional impact of processing affectively toned mental imagery. The use of a concurrent mathematics task during recall of real-life collective trauma has been shown to reduce the emotional impact of recalling the traumatic event in the general population (Engelhard, van den Hout, & Smeets, 2011). Likewise, it has been shown that performance of a concurrent capacity-consuming task reduces the emotional impact of thinking about feared future events in healthy participants (Engelhard, van den Hout, Janssen, & van der Beek, 2010), in students who report high frequency of intrusive future fear imagery (Engelhard, van den Hout, Dek, et al., 2011), and in clinical patients with PTSD (Lilley, Andrade, Turpin, Sabin-Farrell, & Holmes, 2009). Lilley et al. (2009) asked a group of patients with PTSD to generate trauma-related mental imagery while engaging in concurrent lateral eye movements, counting out loud, or performing no concurrent task. Participants in the eye-movement condition reported the lowest levels of imagery vividness, followed by those in the phonological counting condition, while participants in the no-concurrent task control condition reported the highest levels of imagery vividness. The intensity of negative emotion experienced by participants during this procedure followed this exact same function, being lowest in the participants who performed the concurrent eye movement task, intermediate in those who performed the concurrent phonological counting task, and highest in those who performed no concurrent task. Thus, manipulating the vividness of mental imagery through the use of this concurrent task approach served to influence the emotional impact of processing trauma-relevant information.

In addition to voluntarily generated imagery, researchers investigating intrusive flashbacks in PTSD have also begun to evaluate the possibility that capacity-consuming concurrent tasks may also be capable of reducing the frequency and emotional impact of involuntary mental imagery. There is growing evidence that playing the visuospatial computer game “Tetris” following exposure to analogue trauma reduces the frequency of subsequent involuntary memory imagery. Holmes, James, Coode-Bate, and Deeprose (2009) had healthy participants view film clips depicting traumatic scenes, then either play the visuospatial game Tetris after film viewing, or not. Participants used a diary to report intrusive imagery of the film clip content over the following week. Participants who played Tetris after film viewing reported experiencing fewer such imagery intrusions, relative to those who had not. This effect appears to be modality-specific, as playing a predominantly verbal “pub quiz” game after film viewing did reduce subsequent imagery intrusions to the same degree as playing Tetris (Holmes, James, Kilford, & Deeprose, 2010). Subsequent studies have also found Tetris game play to be effective in reducing intrusive memory frequency when memories were reactivated 24 hours after initial film viewing (James et al., 2015). Future research could profitably investigate whether Tetris alleviates imagery intrusions in PTSD symptoms specifically because of visuospatial disruption (James et al., 2015) or as a result of more general working memory taxation (Van den Hout & Engelhard, 2012), as investigators remain divided in their views concerning this issue.

## Section II: Mental Imagery-Evoked Emotional Response in Psychopathology

Mental imagery-evoked emotional responses have been used to study emotional psychopathology, primarily in anxiety research. Evidence has revealed variations in neural and physiological responses across anxiety disorder subtypes. For example, while there is evidence that, overall, anxiety patients exhibit greater amygdala and insula activity during aversive imagery and picture viewing relative to healthy controls, this hyperactivation is more evident in social anxiety disorder and specific phobias compared to PTSD (cf. Etkin & Wager, 2007).

Similarly, during fear relative to neutral memory imagery, specific and social phobics showed greater increases in heart rate and startle reflex response (but not skin conductance) compared to healthy participants (Cuthbert et al., 2003). However, those with panic disorder and PTSD showed hyporeactivity relative to healthy controls, even when baseline heart rate and startle potentiation were taken into account (Cuthbert et al., 2003). Using the same paradigm, McTeague et al. (2010) found that while PTSD patients generally exhibited greater increases in heart rate and startle reflex response (but not in skin conductance) compared to healthy controls, more severe multitrauma PTSD patients showed blunted heart rate, skin conductance, and startle reflex responses compared to single-trauma PTSD patients (McTeague et al., 2010). Indeed, reduced neural fear responding has been linked to the dissociative subtype of PTSD, characterized by symptoms of depersonalization and derealization, symptoms associated with higher trauma severity and chronicity (Lanius, Brand, Vermetten, Frewen, & Spiegel, 2012).

Blunted neurophysiological reactivity to aversive imagery is hypothesized to be attributable to the effects of chronic stress and depression comorbidity, which appears to be more evident in individuals with pervasive anxiety-based disorders rather than focal fear-based phobias (for a review, see Lang & McTeague, 2009).

### Concordance Between Subjective and Physiological Response to Emotional Mental Imagery in Psychopathology

Variations in the level of concordance between subjective emotional distress and physiological arousal during aversive mental imagery have also been investigated as an indicator of emotional psychopathology. This section will review studies that have assessed the level of concordance between self-report, physiological and neural measures.

In healthy volunteers, there is evidence of concordance between self-report and physiological indicators of emotional responding. For example, in a study with healthy volunteers reporting high fear of snakes or public speaking, Lang et al. (1983, Experiment 2) found concordance between self-report and physiological indicators of arousal during fear-imagery, though only when participants had been trained beforehand to focus on their own somatic responses during imagery, and not when participants instead had been trained to focus on visual perceptual information about the stimuli. Similarly, Dalgleish et al. (2013) found concordant variation in subjective reports and physiological indicators (skin conductance and heart rate) of emotional responses during imagery of personal memory imagery of fear, anger, happiness and sadness, in 41 out of 53 participants.

In anxiety disorders, Lang and colleagues found enhanced self-reported distress and heart rate and skin conductance reactivity during imagery of personal phobic stimuli relative to personal non-phobia-related stimuli and standardized stimuli (Cook, Melamed, Cuthbert, McNeil, & Lang, 1988). Interestingly, the congruence between levels of subjective distress and physiological fear response was greatest in participants with simple phobias, followed by those with social phobia, with the least congruence found in those with agoraphobia (Cook et al., 1988). Similarly, McNeil, Vrana, Melamed, Cuthbert, and Lang (1993) assessed aversive imagery in volunteers and clinically anxious patients with simple and social phobias, and found that subjective reports of imagery vividness and emotional distress were positively related to heart rate reactivity in those with simple phobias (e.g., dental surgery) but not in those with social (speech) phobia.

As reviewed in Lang and McTeague (2009), subjective reports of emotional distress in anxiety disorders are not always accompanied by concordant levels of physiological fear response, at least for startle potentiated reflex responses. Whereas exaggerated startle reflex response is found in patients with specific and social phobia during phobic imagery and picture-viewing, blunted startle response is found in patients with more severe anxiety profiles, such as those with agoraphobia, panic disorder, generalized anxiety disorder (GAD), and comorbid depression (Lang & McTeague, 2009). Indeed, physiological response to aversive mental imagery appears to be negatively related to disorder chronicity, severity, and depression-comorbidity, possibly reflecting suppressed defensive reactivity due to long-term stress (McTeague & Lang, 2012).

However, the view that chronic anxiety is associated with negative emotional suppression is inconsistent with the Contrast Avoidance Model of worry (Newman & Llera, 2011). This model postulates that individuals with GAD defensively recruit a perpetuated negative emotional state to emotionally prepare for unexpected negative events. Preliminary evidence in support of this model is provided by the finding that worry-induction in healthy volunteers and GAD patients increases negative emotional states at both subjective and physiological (skin conductance) levels, preventing further increases during exposure to negative imagery (Peasley-Miklus & Vrana, 2000) and film clips (Llera & Newman, 2014). The presence of ceiling effects in negative emotional reactivity to imaginal and real aversive stimuli should be further investigated in GAD and other anxiety-disorder subtypes.

## Section III: The Impact of Mental Imagery on Emotion—A Comparison With Verbal Representation

Section I reviewed evidence that mental-imagery-based representations of emotional information elicit emotional responses at physiological, neurological, and subjective levels. However, as noted earlier, such studies did not contrast mental imagery to an alternative mode of representation of the same information, and therefore it is unknown whether the effects observed during emotional imagery reflect an impact of emotional information processing in general or mental imagery *per se*. This section reviews studies that have attempted to answer this question by contrasting mental imagery to verbal representations of emotional information.

In several studies, Lang and colleagues compared the impact of mental imagery versus verbal information processing of fear-related and neutral sentences (Cuthbert et al., 2003, Vrana et al., 1986). Participants were instructed either to generate mental imagery based on previously memorized fearful sentences, or to verbally repeat the sentences in silence without generating accompanying mental imagery. Results showed significant and sustained heart rate acceleration relative to baseline during mental imagery, but not during verbal repetition of fearful scenarios (Cuthbert et al., 2003, Vrana et al., 1986). This suggests that mental-imagery-based processing of emotional information has a greater impact on emotion than verbal processing.

However, several experimental design issues constrain the interpretation of these results. First, self-reported emotional impact was elicited only following mental imagery of scenarios in Vrana et al. (1986), but not following verbal repetition of scenarios. Thus, it is unclear whether imagery-based processing led to greater subjective emotional response compared to verbal-based processing of the same information (Holmes & Mathews, 2005). Second, due to prior memorization of the cue sentences, participants necessarily engaged in verbal processing of the scenarios prior to completing both mental imagery and verbal repetition conditions. Thus, it is difficult to determine whether differences in emotional response between the imagery and verbal processing conditions simply reflect the recruitment of an additional representational system in the imagery condition (Holmes & Mathews, 2005). Finally, while participants in the imagery condition elaborated on sentence content by simulating them as a personal experience, those in the verbal condition simply repeated the sentences. Thus, it is possible that differences in the level of semantic elaboration between the conditions contributed to differences in observed physiological response.

Following Vrana et al. (1986), a series of studies conducted more controlled comparisons of the subjective emotional impact of mental imagery versus verbal processing of emotionally negative information. A commonly used experimental paradigm has involved exposing participants to scenarios that, while initially ambiguous, ultimately resolved in a manner that produces an emotionally valenced representation of the described situation. Experimenters have contrasted the emotional impact of manipulating whether these representations are imagery-based or verbal in nature. Thus, for example, Holmes and Mathews (2005) instructed healthy participants to either generate imagery or focus on the semantic meaning of 100 such initially ambiguous auditory scenarios that always resolved negatively (“*You are at work when you hear the fire alarms go off. You run to the exit to discover that it is . . . for real*”). Holmes and Mathews (2005) found that participants in the imagery condition reported greater increases in self-reported anxiety as measured using the Spielberger State Anxiety Inventory (STAI-S; Spielberger, Gorsuch, Lushene, Vagg, & Jacobs, 1983) (Experiment 1: *M* = 7.3; Experiment 2: *M* = 5.67), relative to the verbal condition (Experiment 1: *M* = 1.9; Experiment 2: *M* = 1.55).

Importantly, the enhanced emotional impact of mental imagery relative to verbal processing is not restricted to negative material. Using a similar experimental approach, but employing initially ambiguous auditory sentences that always resolved *positively*, Holmes, Mathews, Dalgleish, and Mackintosh (2006) found healthy participants in the mental imagery generation condition reported greater increases in positive affect (*M* = + 7.15, *SD* = 10.30) than those in the verbal condition (*M* = -6.08, *SD* = 9.63), as measured using the Positive and Negative Affect Schedule (PANAS-X; Watson & Clark, 1994). This effect of greater emotional impact of imagery relative to verbal processing has since been replicated in several studies (Holmes, Coughtrey and Connor, 2008, Holmes, Lang and Shah, 2009, Nelis et al., 2012, Pictet et al., 2011).

In addition to transient effects on positive emotion, the impact of positive mental imagery has been shown to persist in the short term. Holmes, Lang, & Shah (2009) asked healthy participants to complete the task described above, and subsequently exposed these participants to a negative mood induction procedure. Participants in the imagery condition experienced a less severe decrement in positive mood following the negative mood induction procedure (PANAS *M* = -4.90, *SD* = 6.88) than did those in the verbal condition (PANAS *M* = -10.90, *SD* = 8.33). Consistent with research on the buffering effects of positive mood on negative mood (Fredrickson, 1998), Holmes, Lang, & Shah (2009) results suggest that positive mental imagery may have a greater potential to buffer the emotional impact of a negative mood induction than does positive verbal thought.

The experimental approach adopted by Holmes and colleagues differs from that employed by Vrana et al. (1986) in two important ways. First, participants in the verbal representation condition were instructed to form meaningful representations of the auditory scenario, requiring them to engage in semantic processing rather than simply engage in verbal repetition of the information, as was the case in Vrana et al.’s approach. Second, subjective emotional response to the information was compared when this was represented in an imagery format and in a verbal format. Thus, while Vrana et al. (1986) were able only to conclude that imagery-based representations exert an impact on subjective emotional experience, Holmes and colleagues have been able to demonstrate that imagery-based representations exert a greater impact on subjective emotional experience than do verbal representations.

In the studies described above, the information that participants were required to represent was always presented in a verbal format. Thus, it could be the case that the greater emotional impact of the imagery condition reflects the fact that the information is represented in two modalities, both verbal and imagery, while in the verbal condition the information is represented in only a single modality (Pictet & Holmes, 2013). To address this issue, an alternative experimental paradigm, the Picture-Word Task (PWT), was developed (Holmes, Mathews, Mackintosh, & Dalgleish, 2008). In the PWT, cue items consist of both a visual image and a negative or benign word caption (e.g., a picture of a person in a lake accompanied by the word *sink* or *swim*). Participants are asked to combine the picture and the word using either imagery (“form a mental image that combines the next picture and word”) or verbal statement (“form a sentence that combines the next picture and the word”). Thus, in this design, the stimulus materials are comprised of image-based and verbal information. Results from the PWT were consistent with the previous finding that imagery-based representation evoked a stronger impact on subjective emotional experience than verbal representation of the same information. Specifically, Holmes, Mathews, Mackintosh, & Dalgleish, 2008 found that using mental imagery to combine negative picture-word cues resulted in greater increases in anxiety (*M* = + 7.06, *SD* = 5.84) than did the use of verbal processing to combine these cues (*M* = + 2.43, *SD* = 3.01); and using mental imagery to combine benign picture-word cues resulted in a greater reduction in anxiety (*M* = -3.81, *SD* = 5.94) than did the use of verbal processing to combine these cues (*M* = + 1.0, *SD* = 2.22).

Therefore, on the basis of the reviewed evidence, it is appropriate to conclude that imagery-based processing of emotional information has a greater impact on subjective emotional experience than does verbal processing of the same information. In relation to Lang’s original postulation that only mental imagery has the capacity to activate physiological and behavioral response systems, evidence from studies on anxiety suggests that more verbal forms of thinking, such as worry, can also impact cardiovascular (Brosschot et al., 2005, Llera and Newman, 2010) and neural activity consistent with a negative emotional response (Oathes et al., 2008). We also note here that worry has been defined as “a chain of thoughts and images, negatively affect-laden and relatively uncontrollable” (Borkovec, Robinson, Pruzinsky, & DePree, 1983).

## Section IV: Harnessing Mental Imagery’s Powerful Emotional Impact in Clinical Practice

When Lang's (1979) bio-informational theory of emotional imagery was developed, behaviorism was the dominant approach within clinical psychology. At this time, mental imagery was used to evoke emotion during imaginal exposure treatment of anxiety disorders to help clients learn new emotional and behavioral responses and train self-control (Goldfried, 1971) via graded imaginal desensitization (Wolpe, 1958) or rapid flooding to feared stimuli (Wolpe, 1973).

Developments in cognitive and social psychology in subsequent decades has shown that an individual’s thoughts can exert a strong influence on his/her behavior and wellbeing. As a result, the content and phenomenology of an individual’s mental imagery-based thoughts have themselves become the subject of analysis and modification (A. T. Beck, 1970, Lazarus, 1968). Building upon Judith Beck’s cognitive therapy imagery formulations (J. S. Beck, 1995), new mental imagery techniques have been brought into the clinic. The following section will briefly review contemporary cognitive and behavioral (CBT) therapeutic techniques that have built upon the legacy of Lang’s early work, which include: (a) imaginal exposure, (b) the direct modification of the content of aversive imagery-based thoughts, (c) the promotion of adaptive imagery, (d) metacognitive reappraisal of imagery, and (e) imagery-based cognitive modification of maladaptive thinking habits.

Imaginal exposure (IE) treatments are still widely used in contemporary clinical practice, with a strong evidence base for treating anxiety disorders ranging from specific phobias (Craske, Antony, & Barlow, 2006), obsessive-compulsive disorder (Abramowitz, Franklin, & Foa, 2002), generalized anxiety disorder (Zinbarg, Craske, & Barlow, 2006), to PTSD (Foa et al., 2007). Seminal advances in mechanistic understandings of fear extinction learning and habituation have been informed by the legacy of Lang’s bio-informational theory and experimental work (e.g., Lang, Melamed, & Hart, 1970). Specifically, it has been proposed that for extinction learning and habituation to occur in imaginal exposure therapy, the imagined feared stimulus itself must evoke fear processing structures in the brain, including both stimulus and response networks (Foa & Kozak, 1986). Evidence for this central mechanism of change comes from the numerous subsequent studies showing that patients who exhibit initial physiological fear responses and subsequent habituation of such responses benefit more from imaginal exposure therapy than those who do not (Craske et al., 1987, Jaycox et al., 1998, Kozak et al., 1988, Mueser et al., 1991).

There is increasing evidence that the efficacy of IE, at times combined with other behavioral or cognitive treatments, is superior to psychopharmacology for OCD (Foa et al., 2005) and PTSD (Van Etten & Taylor, 1998). For GAD, IE-based CBT has been found to be more effective than nondirective and relaxation-based techniques (Borkovec & Costello, 1993). Interestingly, a growing body of research has used virtual reality simulations during exposure therapy to treat a range of anxiety disorders (for reviews see Opriş et al., 2012, Morina et al., 2015). Preliminary evidence suggests that exposure treatments using mental imagery simulations (IE) versus virtual reality simulations of feared stimuli have comparable efficacy for fear of flying (Rus-Calafell, Gutiérrez-Maldonado, Botella, & Baños, 2013) and public speaking (Wallach, Safir, & Bar-Zvi, 2009). In addition to anxiety disorders, researchers have also successfully used IE to reduce intrusive mental imagery in major depression, with promising results in terms of therapeutic benefits (Kandris & Moulds, 2008).

Imagery rescripting techniques designed to directly modify the content of emotion-inducing mental imagery have been developed, helping clients transform their unwanted distressing imagery into more benign forms (Hackmann, Bennett-Levy, & Holmes, 2011). Such techniques have been combined with exposure treatment for a range of disorders, from PTSD and personality disorders (e.g., Arntz and Weertman, 1999, Butler and Holmes, 2009, Long and Quevillon, 2009, Smucker and Dancu, 1999/2005), to snake and social phobia (Hunt and Fenton, 2007, Wild et al., 2007). For PTSD, evidence suggests that imaginal exposure therapy combined with imagery rescripting is more effective than imaginal exposure therapy alone (Arntz et al., 2007, Grunert et al., 2007). Imagery rescripting has also been used to reduce intrusive mental images in healthy populations (Rusch, Grunert, Mendelsohn, & Smucker, 2000) and in people with depression (Brewin et al., 2009). Rescripting of intrusive imagery associated with negative memories has shown initial promise as a stand-alone treatment for major depression (Brewin et al., 2009). For a detailed review of imagery rescripting treatments, see Arntz (2012) and Holmes, Arntz, and Smucker (2007)).

In addition to the development of clinical techniques intended to attenuate negative imagery, clinical investigators have also sought to develop methods of fostering the generation of positive imagery, to help clients build more adaptive relationships between self, others, and the world (Hackmann et al., 2011). Symbolic positive imagery has been used to help clients access adaptive emotional states, in interventions such as Compassion Mind Training (CMT; P. Gilbert, 2009), which was inspired by work on the perfect nurturer image by D. A. Lee (2005). Other positive imagery techniques include guiding clients to construct and road test ideal ways of being in personality disorders (Mooney & Padesky, 2000) or to form mental images of positive scenarios to counter the types of negative beliefs observed in personality disorders, eating disorders, and obsessive-compulsive disorder (Korrelboom, de Jong, Huijbrechts, & Daansen, 2009). Positive imagery can also be used to attenuate or replace negative imagery in vivo. In social anxiety, generating and holding in mind positive images of one’s own appearance, to displace negative images of oneself appearing red and sweaty, has been shown to reduce anxiety and improve social performance (Hirsch, Mathews, Clark, Williams, & Morrison, 2003). Positive imagery has also been shown to function as an effective distractor for chronic pain patients (Fors, Sexton, & Gotestam, 2002).

A third category of imagery-focused interventions aims not to directly alter potentially distressing negative mental imagery but rather to change how the patient thinks about these images, in ways that reduce their emotional impact. Metacognitive strategies aim to down regulate the impact of distressing mental imagery via reappraisal (Gross, 2002). Techniques include encouraging patients to perceive mental images as subjective phenomena (Wells, 2003) rather than meaningful premonitions or signs that one’s mind is out of control (Starr and Moulds, 2006, Williams and Moulds, 2007). Another approach is mindfulness-based cognitive therapy, in which clients are trained to regard their verbal thoughts and mental images as mere passing mental phenomena that do not require a response (Segal, Teasdale, & Williams, 2002).

More recent developments in experimental clinical research have begun to develop mental imagery-focused cognitive training informed by cognitive bias modification (CBM) paradigms. CBM aims to reduce maladaptive imagery styles and boost adaptive imagery via computer-based training (Blackwell et al., 2015, Williams et al., 2015). Such mental imagery training approaches are informed by research that has illuminated psychopathology-linked individual differences in imagery, which will be discussed in more detail with the next section of this review.

Hence, clinical practice is increasingly harnessing the power of mental imagery to facilitate adaptive emotional functioning. The efficacy of such approaches may be further enhanced by future research designed to illuminate the potentially critical role of variability in the strength of the emotional response, observed during imagery in the clinic, in determining treatment outcomes. Given that the elicitation of both subjective and physiological emotional responses during imaginal exposure is a central mechanism of change in the successful treatment of fear, the effectiveness of other treatments that use mental imagery to optimize emotional functioning may also depend on the client’s emotional response during imagery. In our view, research investigating differences between treatment responders compared to nonresponders in the emotional impact of mental imagery may prove fruitful in informing future translational research in this field.

## Section V: Individual Differences in Mental Imagery and Emotional Psychopathology

So far, this review has discussed mental imagery in terms of its capacity to evoke emotional responses, the presence of disorder-linked variations in this emotional response, and the ways that clinical practice has harnessed mental imagery to facilitate emotional processing. However, one relatively neglected aspect of mental imagery that is of potential clinical significance is differences across individuals in the ability and tendency to experience emotional imagery. Here, “ability” is simply defined as the deliberate generation of imagery, and “tendency” as the nondeliberate (i.e., spontaneous) generation of imagery. This section will review research on individual differences in emotional mental imagery tendency and ability in both healthy and clinical populations.

An interesting finding from Lang’s experimental work on the emotional impact of mental imagery is the observation that the level of emotional response experienced during imagery is related to how vividly an individual can generate mental imagery in general (imagery generation ability). Lang and colleagues found that, in healthy participants, heart rate acceleration during fearful imagery was more pronounced in individuals with high imagery ability, relative to those with low imagery ability, as assessed by a self-report questionnaire (Miller et al., 1987). However, such a relationship between self-reported imagery ability and physiological arousal during fear imagery is not always observed. McTeague, Bradley, and Lang (2002) found no association between imagery ability and physiological reactivity during emotional imagery, whether such reactivity was indexed by autonomic responses (heart rate, skin conductance), facial EMG (corrugator and orbicularis), or startle blink reflex.

Relatively few studies have examined individual differences in mental imagery and its relationship to imagery-based treatment outcomes in clinical populations. McNeil et al. (1993) found that physiological reactivity and subjective reports of distress were greater in individuals with higher relative to lower self-reported imagery generation ability. However, this effect was only observed in individuals with specific phobias (e.g., dental), and not in those with social anxiety. In chronic PTSD patients undergoing prolonged imaginal exposure treatment, Rauch, Foa, Furr, and Filip (2004) found that participants’ self-reported anxiety and imagery vividness were correlated, and both decreased significantly during treatment. However, while subjective anxiety change was related to treatment outcome, imagery vividness change was not significantly related to treatment outcome (Rauch et al., 2004). Given these mixed results, it presently remains uncertain how robust a difference there exists in the intensity of emotional responding to imagery, between people who exhibit good and poor imagery ability, and how these differences relate to imagery-based treatment outcomes. The resolution of issue clearly requires further research.

In recent years, researchers have begun to investigate whether bias in the relative ability to generate mental imagery of emotionally negative compared to emotionally positive scenarios may be associated with emotional psychopathology. Several studies have used the Prospective Imagery Task (PIT; Holmes, Lang, et al., 2008, Stöber, 2000) to assess imagery vividness. In this task, participants are required to generate mental imagery cued by negative and positive sentences depicting self-referential future scenarios (e.g., negative scenario: “You will be the victim of crime”; positive scenario: “You will have lots of energy and enthusiasm”). Results from such studies indicate that, compared to healthy controls, individuals with depressed mood (Holmes, Lang, Moulds, & Steele, 2008), and those with a diagnosis of clinical depression or anxiety (Morina, Deeprose, Pusowski, Schmid, & Holmes, 2011) report lower imagery vividness (i.e., visual clarity) for positive scenarios. Participants with depressed mood (Holmes, Lang, Moulds, & Steele, 2008) and anxiety disorders (Morina et al., 2011) also report greater vividness of imagery for negative scenarios compared to healthy controls, though this latter effect is not observed in clinically depressed participants (Morina et al., 2011).

In addition, researchers have begun to move beyond cross-sectional data and to examine temporal relationships between imagery vividness and emotional psychopathology. Blackwell et al. (2015) conducted a randomized controlled trial in which 150 depressed adults received either a positive imagery intervention involving repeated generation of mental imagery involving pleasant daily activities, or a nonimagery “sham training” control condition, both delivered via the Internet over 4 weeks. Results indicate that the more vividly participants in the positive imagery condition could imagine the positive training scenarios at baseline, the greater their reduction in depression symptoms over the course of the intervention (Blackwell et al., 2015). Future studies should more directly assess the relationship between changes in imagery vividness and changes in symptomology.

Researchers have also started to explore disorder-linked individual differences in the tendency to spontaneously experience emotional mental imagery in daily life. Negative self-concept in unselected undergraduate students has been found to be positively associated with the frequency of retrospectively reported negative spontaneous cognitions, consisting predominantly of imagery-based memories of past negative experiences (Krans, de Bree, & Moulds, 2015). It has also been shown that, compared to formerly-depressed and never-depressed individuals, depressed individuals report experiencing spontaneous imagery-based memories, which were predominantly negative in content, as more vivid, evoking greater subjective distress, and causing greater disruption to daily life (Newby & Moulds, 2011).

Future emotional psychopathology-linked individual differences research may benefit from obtaining convergent measures of emotional response during mental imagery, such as measures of physiology and hemodynamic neural activity. In addition to indices of emotional response during imagery, electroencephalography (EEG) measures of cortical brain electrical activity may provide indices of the level of cognitive resources expended during imagery as an additional measure of the impact of emotional imagery. One recent study from Lang and colleagues examining alpha-band activity during emotional mental imagery suggests EEG may provide an objective measure of neural activity involved in semantic, motor, and perceptual representations of emotional stimuli. Bartsch, Hamuni, Miskovic, Lang, and Keil (2015) found that while mental imagery elicited by pleasant and unpleasant verbs prompted equivalent levels of alpha amplitude, both were higher than that observed during imagery of emotionally neutral verbs. Alpha-band (8-12 Hz) activity is associated with internally directed attention and may be used to index individual differences in emotional imagery-related brain activity in translational and clinical research.

Could it be that individual differences in imagery-based processing of verbal information causally underpins vulnerability to emotional psychopathology, and/or functionally contributes to its maintenance? So far, research linking individual differences in emotional mental imagery generation and emotional psychopathology has been correlational in nature**.** More experimental research is required to investigate if and how the ability and tendency to experience emotional mental imagery is causally related to emotional wellbeing and dysfunction. Several mechanisms have been proposed and require further investigation. One hypothesis is that mental imagery acts as an emotional amplifier and exacerbates states of mood instability, such as in bipolar disorder (Holmes, Geddes, Colom, & Goodwin, 2008). From a treatment perspective, enhancing the ability to mentally simulate positive scenarios may causally relate to improved emotional functioning via cognitive and behavioral mechanisms such as enhanced negative mood repair, and/or increased anticipatory pleasure and approach motivation for daily activities. The outcomes of such future research will inform how best to exploit our increasing understanding of individual differences in mental imagery in ways that can enhance the efficacy of our therapeutic interventions for clinical anxiety and depression.

## Section VI: Neglected Insights From Lang’s Work on Mental Imagery Emotional Response Training

A critical yet largely overlooked finding from Lang, 1977, Lang, 1979 experimental work is the demonstration that the emotional impact of imagery can be modulated via training. Lang’s psychophysiology studies provided reliable evidence that physiological arousal during mental imagery of fearful scenes can be increased in two ways. One is via the inclusion of somatovisceral response information in the verbal scripts used to cue imagery (e.g., “your heart is pounding”). The other is via the positive reinforcement of participants’ verbal reports of mental imagery containing somatovisceral response during an initial training phase (e.g., “I felt myself running down the hill fast . . . my heart was beating”). Several studies by Lang and colleagues have demonstrated that such imagery training amplified participants’ ability to elicit physiological responses during imagery (Lang et al., 1980, Lang et al., 1983, Miller et al., 1987). As discussed earlier, the effectiveness of imaginal exposure therapy rests partly on the presence of initial emotional arousal to the imaginal feared stimuli. Thus, training that enhances emotional response during negative imagery may improve fear-extinction treatment outcomes.

Furthermore, training that can increase the emotional impact of mental imagery may serve to enhance the effectiveness of positive imagery interventions for clinical patients who exhibit deficits in positive imagery. As described earlier, the cognitive bias modification approach has recently been combined with mental imagery training to target interpretation bias in both mood and anxiety disorders. Imagery-based interpretation bias modification (IBM) training involves repeatedly generating imagery in response to verbal sentences and picture-word pairs depicting hypothetical everyday scenarios that resolve positively. Experimental studies have shown that imagery-based IBM training of this nature improves mood to a greater degree than does verbal-based IBM training (Holmes, Lang and Shah, 2009, Holmes et al., 2006; T. Lang, Blackwell, Harmer, Davison, & Holmes, 2012). To date, several randomized-controlled clinical trials have been conducted, with mixed preliminary findings. While results from two such trials indicate positive imagery IBM training to be a promising and accessible Internet intervention for improving mood and alleviating symptoms in depression (Williams et al., 2013, Williams et al., 2015), another trial found no support for the positive IBM imagery condition being better than a non-imagery-focused, noninterpretation training, control condition (Blackwell et al., 2015). It seems reasonable to suppose that the efficacy of this imagery-based IBM training would be further enhanced by increasing the positive emotional impact of the trained pattern of positive imagery. This objective may be served by integrating the somatovisceral response amplification techniques, developed by Lang and colleagues, into imagery-based IBM programs designed to increase the occurrence of positive imagery, which represents an important avenue for future research in this area.

## Section VII: Conclusion and Future Directions

Charting the progress of emotional mental imagery research across the past five decades since Lang’s (1977) seminal paper has clearly illustrated the major impact of his bio-informational theory and experimental research on this field. Following Lang’s footsteps, both experimental and translational researchers have continued to be fascinated by the special relationship that exists between mental imagery and emotion.

In emphasizing mental imagery’s capacity to activate perceptual stimulus representations as well as physiological and behavioral responses, Lang’s theory and experimental work provided crucial insights into the field of behavior therapy. Specifically, physiological fear response during imagery of feared stimuli has been identified as the marker of successful learning during imaginal exposure and habituation. In light of this finding, evidence reviewed in Section I showing blunted physiological fear response in patients suffering more chronic anxiety and mood dysfunction represents a serious concern in the clinical field. Specifically, it suggests that such individuals may require additional support to enhance physiological responding in order to maximally benefit from the corrective effects of imaginal (or in vivo) exposure therapy. Given that the success of imaginal exposure therapy in facilitating fear-extinction hinges upon the patient exhibiting initial physiological arousal to the imagined fear stimuli, the efficacy of other CBT treatments using mental imagery to facilitate emotional processing may also be enhanced by taking steps to ensure that emotional responses to mental imagery are maximized. Future clinical translational and treatment efficacy research may well benefit from using training techniques pioneered by Lang to keep affect “hot” during critical moments in therapy.

Recent individual differences research has begun to illuminate how bias in the relative ability to voluntarily generate emotional imagery, and bias in the tendency to spontaneously experience emotional imagery, differentiates those experiencing emotional disturbance from healthy individuals. This line of experimental research has important clinical implications, as an excess of unwanted emotional mental imagery requires therapeutic alteration. Further, the inability to generate positive imagery, or inability to respond to negative imagery, has treatment implications for depression and exposure therapy. Particularly promising is experimental research that investigates how the relationship between mental imagery and emotion can be exploited for clinical benefit via cognitive bias modification training. Future research on positive mental imagery training could be enhanced by incorporating Lang’s method of emotional imagery response training, using imagery-eliciting scripts that contain both stimulus and response information, combined with training procedures that positively reinforce emotional responding during mental imagery. Furthermore, measuring both subjective and neurophysiological indices of emotional response during positive mental imagery training may provide additional information in identifying treatment responders in future research.

Nearly 40 years ago, Lang’s seminal work on emotional imagery laid the foundation for a new era of experimental and clinical research on this fascinating topic. While subsequent researchers have been able to build upon this firm foundation in ways that have greatly expanded knowledge and understanding, it is remarkable that Lang’s original questions, and the findings produced by Lang and his collaborators, have remained of such central importance within the burgeoning contemporary literature. We are confident that this will continue to be the case across the years that lie ahead, and we are honored to have been given this opportunity to celebrate, and pay tribute to, Lang’s extraordinary scientific and clinical legacy.

## Conflict of Interest Statement

The authors declare that there are no conflicts of interest.

## References

[bb0005] Abramowitz J.S., Franklin M.E., Foa E.B. (2002). Empirical status of cognitive-behavioral therapy for obsessive-compulsive disorder: A meta-analytic review. Romanian Journal of Cognitive and Behavioral Psychotherapies.

[bb0010] Andrade J., Kavanagh D.J., Baddeley A.D. (1997). Eye-movements and visual imagery: A working memory approach to the treatment of post-traumatic stress disorder. British Journal of Clinical Psychology.

[bb0015] Arntz A. (2012). Imagery rescripting as a therapeutic technique: Review of clinical trials, basic studies, and research agenda. Journal of Experimental Psychopathology.

[bb0020] Arntz A., Tiesema M., Kindt M. (2007). Treatment of PTSD: A comparison of imaginal exposure with and without imagery rescripting. Journal of Behavior Therapy and Experimental Psychiatry.

[bb0025] Arntz A., Weertman A. (1999). Treatment of childhood memories: Theory and practice. Behaviour Research and Therapy.

[bb0030] Bartsch F., Hamuni G., Miskovic V., Lang P.J., Keil A. (2015). Oscillatory brain activity in the alpha range is modulated by the content of word‐prompted mental imagery. Psychophysiology.

[bb0035] Beck A.T. (1970). Cognitive therapy: Nature and relation to behavior therapy. Behavior Therapy.

[bb0040] Beck J.S. (1995). Cognitive therapy: Basics and beyond.

[bb0045] Blackwell S.E., Browning M., Mathews A., Pictet A., Welch J., Davies J., . . ., Holmes E.A. (2015). Positive imagery-based cognitive bias modification as a web-based treatment tool for depressed adults: A randomized controlled trial. Clinical Psychological Science.

[bb0050] Borkovec T.D., Costello E. (1993). Efficacy of applied relaxation and cognitive-behavioral therapy in the treatment of generalized anxiety disorder. Journal of Consulting and Clinical Psychology.

[bb0055] Borkovec T.D., Robinson E., Pruzinsky T., DePree J.A. (1983). Preliminary exploration of worry some characteristics and processes. Behaviour Research and Therapy.

[bb0060] Brewin C.R., Holmes E.A. (2003). Psychological theories of posttraumatic stress disorder. Clinical Psychology Review.

[bb0065] Brewin C.R., Wheatley J., Patel T., Fearon P., Hackmann A., Wells A., . . ., Myers S. (2009). Imagery rescripting as a brief stand-alone treatment for depressed patients with intrusive memories. Behaviour Research and Therapy.

[bb2000] Brosschot J.F., Pieper S., Thayer J.F. (2005). Expanding stress theory: Prolonged activation and perseverative cognition. *Psychoneuroendocrinology*.

[bb0070] Butler G., Holmes E.A., Stopa L. (2009). Imagery and the self following childhood trauma: Observations concerning the use of drawings and external images. Imagery and the threatened self: Perspectives on mental imagery and the self in cognitive therapy.

[bb0075] Cook E.W., Melamed B.G., Cuthbert B.N., McNeil D.W., Lang P.J. (1988). Emotional imagery and the differential diagnosis of anxiety. Journal of Consulting and Clinical Psychology.

[bb0080] Cook E.W., Hawk L.W., Davis T.L., Stevenson V.E. (1991). Affective individual differences and startle reflex modulation. Journal of Abnormal Psychology.

[bb0085] Costa V.D., Lang P.J., Sabatinelli D., Versace F., Bradley M.M. (2010). Emotional imagery: assessing pleasure and arousal in the brain's reward circuitry. Human Brain Mapping.

[bb0090] Craig A.B. (2009). How do you feel—now? The anterior insula and human awareness. Nature Reviews Neuroscience.

[bb0095] Craske M.G., Antony M.M., Barlow D.H. (2006). Mastering your fears and phobias: Therapist guide.

[bb0100] Craske M.G., Sanderson W.C., Barlow D.H. (1987). How do desynchronous response systems relate to the treatment of agoraphobia: A follow-up evaluation. Behaviour Research and Therapy.

[bb0105] Cuthbert B.N., Lang P.J., Strauss C., Drobes D., Patrick C.J., Bradley M.M. (2003). The psychophysiology of anxiety disorder: Fear memory imagery. Psychophysiology.

[bb0110] Dadds M.R., Bovbjerg D.H., Redd W.H., Cutmore T.R.H. (1997). Imagery in human classical conditioning. Psychological Bulletin.

[bb0115] Dalgleish T., Navrady L., Bird E., Hill E., Dunn B.D., Golden A. (2013). Method-of-loci as a mnemonic device to facilitate access to self-affirming personal memories for individuals with depression. Clinical Psychological Science.

[bb0120] Damasio A.R., Grabowski T.J., Bechara A., Damasio H., Ponto L.L., Parvizi J., Hichwa R.D. (2000). Subcortical and cortical brain activity during the feeling of self-generated emotions. Nature Neuroscience.

[bb0125] Ehlers A., Hackmann A., Steil R., Clohessy S., Wenninger K., Winter H. (2002). The nature of intrusive memories after trauma: The warning signal hypothesis. Behaviour Research and Therapy.

[bb0130] Engelhard I.M., van den Hout M.A., Dek E.C.P., Giele C.L., van der Wielen J., Reijnen M.J., van Roij B. (2011). Reducing vividness and emotional intensity of recurrent ''flashforwards'' by taxing working memory: An analogue study. Journal of Anxiety Disorders.

[bb0135] Engelhard I.M., van den Hout M.A., Janssen W.C., van der Beek J. (2010). Eye movements reduce vividness and emotionality of "flashforwards". Behaviour Research and Therapy.

[bb0140] Engelhard I.M., van den Hout M.A., Smeets M.A.M. (2011). Taxing working memory reduces vividness and emotional intensity of images about the Queen's Day tragedy. Journal of Behavior Therapy and Experimental Psychiatry.

[bb0145] Etkin A., Wager T.D. (2007). Functional neuroimaging of anxiety: a meta-analysis of emotional processing in PTSD, social anxiety disorder, and specific phobia. American Journal of Psychiatry.

[bb0150] Foa E.B., Hembree E.A., Rothbaum B.O. (2007). Prolonged exposure therapy for PTSD: Emotional processing of traumatic experiences.

[bb0155] Foa E.B., Kozak M.J. (1986). Emotional processing of fear: Exposure to corrective information. Psychological Bulletin.

[bb0160] Foa E.B., Liebowitz M.R., Davies S., Campeas R., Franklin M.E., Huppert J.D., . . ., Tu X (2005). Randomized, placebo-controlled trial of exposure and ritual prevention, clomipramine, and their combination in the treatment of obsessive-compulsive disorder. American Journal of Psychiatry.

[bb0165] Fors E.A., Sexton H., Gotestam K.G. (2002). The effect of guided imagery and amitriptyline on daily fibromyalgia pain: a prospective, randomized, controlled trial. Journal of Psychiatric Research.

[bb0170] Fredrickson B.L. (1998). What good are positive emotions?. Review of General Psychology.

[bb0175] Galton F. (1880). Statistics of mental imagery. Mind.

[bb0180] Gilbert D.T., Wilson T.D. (2007). Prospection: Experiencing the future. Science.

[bb0185] Gilbert P. (2009). Introducing compassion-focused therapy. Advances in Psychiatric Treatment.

[bb0190] Goldfried M.R. (1971). Systematic desensitization as training in self-control. Journal of Consulting and Clinical Psychology.

[bb0195] Gross J.J. (2002). Emotion regulation: Affective, cognitive, and social consequences. Psychophysiology.

[bb0200] Gross J.J., Barrett L.F. (2011). Emotion generation and emotion regulation: One or two depends on your point of view. Emotion Review.

[bb0205] Grunert B.K., Weis J.M., Smucker M.R., Christianson H.F. (2007). Imagery rescripting and reprocessing therapy after failed prolonged exposure for post-traumatic stress disorder following industrial injury. Journal of Behavior Therapy and Experimental Psychiatry.

[bb0210] Hackmann A., Bennett-Levy J., Holmes E.A. (2011). Oxford guide to imagery in cognitive therapy.

[bb0215] Hirsch C.R., Holmes E.A. (2007). Mental imagery in anxiety disorders. Psychiatry.

[bb0220] Hirsch C.R., Mathews A., Clark D.M., Williams R., Morrison J. (2003). Negative self-imagery blocks inferences. Behaviour Research and Therapy.

[bb0225] Holmes E.A., Arntz A., Smucker M.R. (2007). Imagery rescripting in cognitive behaviour therapy: Images, treatment techniques and outcomes. Journal of Behavior Therapy and Experimental Psychiatry.

[bb0230] Holmes E.A., Coughtrey A.E., Connor A. (2008). Looking at or through rose-tinted glasses? Imagery perspective and positive mood. Emotion.

[bb0235] Holmes E.A., Crane C., Fennell M.J.V., Williams J.M.G. (2007). Imagery about suicide in depression — "Flash-forwards"?. Journal of Behavior Therapy and Experimental Psychiatry.

[bb3000] Holmes E.A., Geddes J.R., Colom F., Goodwin G.M. (2008). Mental imagery as an emotional amplifier: Application to bipolar disorder. Behaviour Research and Therapy.

[bb0240] Holmes E.A., James E.L., Coode-Bate T., Deeprose C. (2009). Can playing the computer game "Tetris'' reduce the build-up of flashbacks for trauma? A proposal from cognitive science. PLoS ONE.

[bb0245] Holmes E.A., James E.L., Kilford E.J., Deeprose C. (2010). Key steps in developing a cognitive vaccine against traumatic flashbacks: Visuospatial Tetris versus verbal Pub Quiz. PLoS ONE.

[bb0250] Holmes E.A., Lang T.J., Moulds M.L., Steele A.M. (2008). Prospective and positive mental imagery deficits in dysphoria. Behaviour Research and Therapy.

[bb0255] Holmes E.A., Lang T.J., Shah D.M. (2009). Developing interpretation bias modification as a 'cognitive vaccine' for depressed mood—Imagining positive events makes you feel better than thinking about them verbally. Journal of Abnormal Psychology.

[bb0260] Holmes E.A., Mathews A. (2005). Mental imagery and emotion: A special relationship?. Emotion.

[bb0265] Holmes E.A., Mathews A. (2010). Mental imagery in emotion and emotional disorders. Clinical Psychology Review.

[bb0270] Holmes E.A., Mathews A., Dalgleish T., Mackintosh B. (2006). Positive interpretation training: effects of mental imagery versus verbal training on positive mood. Behavior Therapy.

[bb0275] Holmes E.A., Mathews A., Mackintosh B., Dalgleish T. (2008). The causal effect of mental imagery on emotion assessed using picture-word cues. Emotion.

[bb0280] Hunt M.G., Fenton M. (2007). Imagery rescripting versus in vivo exposure in the treatment of snake fear. Journal of Behavior Therapy and Experimental Psychiatry.

[bb0285] James E.L., Bonsall M.B., Hoppitt L., Tunbridge E.M., Geddes J.R., Milton A.L., Holmes E.A. (2015). Computer game play reduces intrusive memories of experimental trauma via reconsolidation update mechanisms. Psychological Science.

[bb0290] Jaycox L.H., Foa E.B., Morral A.R. (1998). Influence of emotional engagement and habituation on exposure therapy for PTSD. Journal of Consulting and Clinical Psychology.

[bb0295] Kandris E., Moulds M.L. (2008). Can imaginal exposure reduce intrusive memories in depression? A case study. Cognitive Behaviour Therapy.

[bb0300] Kavanagh D.J., Freese S., Andrade J., May J. (2001). Effects of visuospatial tasks on desensitization to emotive memories. British Journal of Clinical Psychology.

[bb0305] Kim S.E., Kim J.W., Kim J.J., Jeong B.S., Choi E.A., Jeong Y.G. (2007). The neural mechanism of imagining facial affective expression. Brain Research.

[bb0310] Korrelboom K., de Jong M., Huijbrechts I., Daansen P. (2009). Competitive memory training (COMET) for treating low self-esteem in patients with eating disorders: A randomized clinical trial. Journal of Consulting and Clinical Psychology.

[bb0315] Kosslyn S.M. (1981). The medium and the message in mental-imagery: A theory. Psychological Review.

[bb0320] Kosslyn S.M., Ganis G., Thompson W.L. (2001). Neural foundations of imagery. Nature Reviews Neuroscience.

[bb0325] Kosslyn S.M., Shin L.M., Thompson W.L., McNally R.J., Rauch S.L., Pitman R.K., Alpert N.M. (1996). Neural effects of visualizing and perceiving aversive stimuli: A PET investigation. Neuroreport.

[bb0330] Kozak M.J., Foa E.B., Steketee G. (1988). Process and outcome of exposure treatment with obsessive-compulsives: Psychophysiological indicators of emotional processing. Behavior Therapy.

[bb0335] Krans J., de Bree J., Moulds M.L. (2015). Involuntary cognitions in everyday life: Exploration of type, quality, content and function. Frontiers in Psychiatry.

[bb0340] Kreiman G., Koch C., Fried I. (2000). Imagery neurons in the human brain. Nature.

[bb0345] Kuyken W., Brewin C.R. (1994). Intrusive memories of childhood abuse during depressive episodes. Behaviour Research and Therapy.

[bb0350] Lang P.J. (1977). Imagery in therapy: An information processing analysis of fear. Behavior Therapy.

[bb0355] Lang P.J. (1979). A bio-informational theory of emotional imagery. Psychophysiology.

[bb0360] Lang P.J. (1987). Image as action: A reply to Watts and Blackstock. Cognition & Emotion.

[bb0365] Lang P.J., Kozak M.J., Miller G.A., Levin D.N., McLean A. (1980). Emotional imagery: Conceptual structure and pattern of somato-visceral response. Psychophysiology.

[bb0370] Lang P.J., Levin D.N., Miller G.A., Kozak M.J. (1983). Fear behavior, fear imagery, and the psychophysiology of emotion: the problem of affective response integration. Journal of Abnormal Psychology.

[bb0375] Lang P.J., McTeague L.M. (2009). The anxiety disorder spectrum: Fear imagery, physiological reactivity, and differential diagnosis. Anxiety, Stress, & Coping.

[bb0380] Lang P.J., Melamed B.G., Hart J. (1970). A psychophysiological analysis of fear modification using an automated desensitization procedure. Journal of Abnormal Psychology.

[bb0385] Lang T.J., Blackwell S.E., Harmer C.J., Davison P., Holmes E.A. (2012). Cognitive bias modification using mental imagery for depression: developing a novel computerized intervention to change negative thinking styles. European Journal of Personality.

[bb0390] Lanius R.A., Brand B., Vermetten E., Frewen P.A., Spiegel D. (2012). The dissociative subtype of posttraumatic stress disorder: Rationale, clinical and neurobiological evidence, and implications. Depression and Anxiety.

[bb0395] Lazarus A.A. (1968). Variations in desensitization therapy. Psychotherapy: Theory, Research & Practice.

[bb0400] Lee D.A., Gilbert I.P. (2005). The perfect nurturer: A model to develop a compassionate mind within the context of cognitive therapy. Compassion: Conceptualisations, Research and Use in Psychothrapy.

[bb0405] Lee S., Ruiz S., Caria A., Veit R., Birbaumer N., Sitaram R. (2011). Detection of cerebral reorganization induced by real-time fMRI feedback training of insula activation a multivariate investigation. Neurorehabilitation and Neural Repair.

[bb0410] Lewis D.E., O'Reilly M.J., Khuu S.K., Pearson J. (2013). Conditioning the mind's eye: Associative learning with voluntary mental imagery. Clinical Psychological Science.

[bb0415] Lilley S.A., Andrade J., Turpin G., Sabin-Farrell R., Holmes E.A. (2009). Visuospatial working memory interference with recollections of trauma. British Journal of Clinical Psychology.

[bb4000] Llera S.J., Newman M.G. (2010). Effects of worry on physiological and subjective reactivity to emotional stimuli in generalized anxiety disorder and nonanxious control participants. Emotion.

[bb0420] Llera S.J., Newman M.G. (2014). Rethinking the role of worry in generalized anxiety disorder: evidence supporting a model of emotional contrast avoidance. Behavior Therapy.

[bb0425] Long M.E., Quevillon R. (2009). Imagery rescripting in the treatment of posttraumatic stress disorder. Journal of Cognitive Psychotherapy.

[bb0430] McNeil D.W., Vrana S.R., Melamed B.G., Cuthbert B.N., Lang P.J. (1993). Emotional imagery in simple and social phobia: fear versus anxiety. Journal of Abnormal Psychology.

[bb0435] McTeague L.M., Bradley M.M., Lang P.J. (2002). Creating a mental image: Is a picture worth a thousand words?. Psychophysiology.

[bb0440] McTeague L.M., Lang P.J. (2012). The anxiety spectrum and the reflex physiology of defense: from circumscribed fear to broad distress. Depression and Anxiety.

[bb0445] McTeague L.M., Lang P.J., Laplante M.C., Cuthbert B.N., Shumen J.R., Bradley M.M. (2010). Aversive imagery in posttraumatic stress disorder: trauma recurrence, comorbidity, and physiological reactivity. Biological Psychiatry.

[bb0450] Miller G.A., Levin D.N., Kozak M.J., Cook E.W., McLean A., Lang P.J. (1987). Individual differences in imagery and the psychophysiology of emotion. Cognition and Emotion.

[bb0455] Mooney K.A., Padesky C.A. (2000). Applying client creativity to current problems: Constructing possibilities and tolerating doubt. Journal of Cognitive Psychotherapy: An International Quarterly.

[bb0460] Morewedge C.K., Huh Y.E., Vosgerau J. (2010). Thought for food: imagined consumption reduces actual consumption. Science.

[bb0465] Morina N., Deeprose C., Pusowski C., Schmid M., Holmes E.A. (2011). Prospective mental imagery in patients with major depressive disorder or anxiety disorders. Journal of Anxiety Disorders.

[bb6900] Morina N., Ijntema H., Meyerbröker K., Emmelkamp P.M. (2015). Can virtual reality exposure therapy gains be generalized to real-life? A meta-analysis of studies applying behavioral assessments. Behaviour Research and Therapy.

[bb0470] Moulton S.T., Kosslyn S.M. (2009). Imagining predictions: Mental imagery as mental emulation. Philosophical Transactions of The Royal Society B: Biological Sciences.

[bb0475] Mueser K.T., Yarnold P.R., Foy D.W. (1991). Statistical analysis for single-case designs evaluating outcome of imaginal exposure treatment of chronic PTSD. Behavior Modification.

[bb0480] Murphy F.C., Nimmo-Smith I., Lawrence A.D. (2003). Functional neuroanatomy of emotion: A meta-analysis. Cognitive, Affective & Behavioral Neuroscience.

[bb0485] Nelis S., Vanbrabant K., Holmes E.A., Raes F. (2012). Greater positive affect change after mental imagery than verbal thinking in a student sample. Journal of Experimental Psychopathology.

[bb2900] Newby J.M., Moulds M.L. (2011). Characteristics of intrusive memories in a community sample of depressed, recovered depressed and never-depressed individuals. Behaviour Research and Therapy.

[bb0495] Newman M.G., Llera S.J. (2011). A novel theory of experiential avoidance in generalized anxiety disorder: A review and synthesis of research supporting a Contrast Avoidance Model of worry. Clinical Psychological Review.

[bb1500] Oathes D.J., Ray W.J., Yamasaki A.S., Borkovec T.D., Castonguay L.G., Newman M.G., Nitschke J. (2008). Worry, generalized anxiety disorder, and emotion: Evidence from the EEG gamma band. Biological psychology.

[bb6500] Opriş D., Pintea S., García‐Palacios A., Botella C., Szamosközi Ş, David D. (2012). Virtual reality exposure therapy in anxiety disorders: A quantitative meta‐analysis. Depression and anxiety.

[bb0500] Partiot A., Grafman J., Sadato N., Wachs J., Hallett M. (1995). Brain activation during the generation of nonemotional and emotional plans. Neuroreport.

[bb0505] Pearson J., Naselaris T., Holmes E.A., Kosslyn S.M. (2015). Mental imagery: functional mechanisms and clinical applications. Trends in Cognitive Sciences.

[bb0510] Peasley-Miklus C., Vrana S.R. (2000). Effect of worrisome and relaxing thinking on fearful emotional processing. Behaviour Research and Therapy.

[bb0515] Phan K.L., Wager T., Taylor S.F., Liberzon I. (2002). Functional neuroanatomy of emotion: a meta-analysis of emotion activation studies in PET and fMRI. Neuroimage.

[bb0520] Pictet A., Coughtrey A.E., Mathews A., Holmes E.A. (2011). Fishing for happiness: The effects of positive imagery on interpretation bias and a behavioral task. Behaviour Research and Therapy.

[bb0525] Pictet A., Holmes E.A., Rimé B., Mesquita B., Hermans D. (2013). The powerful impact of mental imagery in changing emotion. Changing emotions.

[bb0530] Pylyshyn Z.W. (1973). What the mind's eye tells the mind's brain: A critique of mental imagery. Psychological Bulletin.

[bb0535] Rauch S.A., Foa E.B., Furr J.M., Filip J.C. (2004). Imagery vividness and perceived anxious arousal in prolonged exposure treatment for PTSD. Journal of Traumatic Stress.

[bb3500] Rus-Calafell M., Gutiérrez-Maldonado J., Botella C., Baños R.M. (2013). Virtual reality exposure and imaginal exposure in the treatment of fear of flying: A pilot study. Behavior Modification.

[bb0540] Rusch M.D., Grunert B.K., Mendelsohn R.A., Smucker M.R. (2000). Imagery rescripting for recurrent, distressing images. Cognitive and Behavioral Practice.

[bb0545] Sabatinelli D., Bradley M.M., Lang P.J., Costa V.D., Versace F. (2007). Pleasure rather than salience activates human nucleus accumbens and medial prefrontal cortex. Journal of Neurophysiology.

[bb0550] Schacter D.L., Addis D.R., Buckner R.L. (2008). Episodic simulation of future events concepts, data, and applications. New York Academy of Sciences.

[bb0555] Schaefer A., Collette F., Philippot P., Van der Linden M., Laureys S., Delfiore G., Salmon E. (2003). Neural correlates of “hot” and “cold” emotional processing: a multilevel approach to the functional anatomy of emotion. NeuroImage.

[bb0560] Segal Z.V., Teasdale J.D., Williams J.M.G. (2002). Mindfulness-based cognitive therapy for depression.

[bb0565] Sharot T., Riccardi A.M., Raio C.M., Phelps E.A. (2007 Nov. 1). Neural mechanisms mediating optimism bias. Nature.

[bb0570] Sheehan P.W. (1967). A shortened form of Betts questionnaire upon mental imagery. Journal of Clinical Psychology.

[bb0575] Shin L.M., Dougherty D.D., Orr S.P., Pitman R.K., Lasko M., Macklin M.L., . . ., Rauch S.L. (2000). Activation of anterior paralimbic structures during guilt- related script-driven imagery. Biological Psychiatry.

[bb0580] Smucker M.R., Dancu C.V. (1999/2005). Cognitive-behavioral treatment for adult survivors of childhood trauma: Imagery rescripting and reprocessing.

[bb0585] Spielberger C.D., Gorsuch R.L., Lushene R., Vagg P.R., Jacobs G.A. (1983). Manual for State-Trait Anxiety Inventory.

[bb0590] Starr S., Moulds M. (2006). The role of negative interpretations of intrusive memories in depression. Journal of Affective Disorders.

[bb0595] Stöber J. (2000). Prospective cognitions in anxiety and depression: Replication and methodological extension. Cognition & Emotion.

[bb0600] Suddendorf T., Corballis M.C. (2007). The evolution of foresight: what is mental time travel, and is it unique to humans?. Behavioral and Brain Sciences.

[bb0605] Taylor S.E., Pham L.B., Rivkin I.D., Armor D.A. (1998). Harnessing the imagination: Mental stimulation, self-regulation, and coping. The American Psychologist.

[bb0610] Van den Hout M.A., Engelhard I.M. (2012). How does EMDR work?. Journal of Experimental Psychopathology.

[bb0615] Van Diest I., Winters W., Devriese S., Vercamst E., Han J.N., Van de Woestijne K.P., Van den Bergh O. (2001). Hyperventilation beyond fight/flight: Respiratory responses during emotional imagery. Psychophysiology.

[bb0620] Van Etten M.L., Taylor S. (1998). Comparative efficacy of treatments for post-traumatic stress disorder: a meta-analysis. Clinical Psychology & Psychotherapy.

[bb0625] Vanhaudenhuyse A., Bruno M., Bredart S., Plenevaux A., Laureys S. (2007). The challenge of disentangling reportability and phenomenal consciousness in post-comatose states. Behavioral and Brain Sciences.

[bb0630] Vrana S.R. (1995). Emotional Modulation of Skin-Conductance and Eyeblink Responses to a Startle Probe. Psychophysiology.

[bb0635] Vrana S.R., Cuthbert B.N., Lang P.J. (1986). Fear imagery and text-processing. Psychophysiology.

[bb0640] Vrana S.R., Lang P.J. (1990). Fear imagery and the startle-probe reflex. Journal of Abnormal Psychology.

[bb5500] Wallach H.S., Safir M.P., Bar-Zvi M. (2009). Virtual reality cognitive behavior therapy for public speaking anxiety A randomized clinical trial. Behavior Modification.

[bb0645] Watson D., Clark L.A. (1994). The PANAS-X - Manual for the Positive and Negative Affect Schedule - Expanded form.

[bb0650] Watts F.N., Williams J.M.G., Watts F.N., MacLeod C., Mathews A. (1997). Thoughts and Images. Cognitive Psychology and Emotional Disorders.

[bb0655] Weerts T.C., Lang P.J. (1978). Psychophysiology of fear imagery: Differences between focal phobia and social performance anxiety. Journal of Consulting and Clinical Psychology.

[bb0660] Wells A. (2003). Emotional disorders and metacognition: Innovative cognitive therapy. British Journal of Clinical Psychology.

[bb0665] Wild J., Hackmann A., Clark D.M. (2007). When the present visits the past: Updating traumatic memories in social phobia. Journal of Behavior Therapy and Experimental Psychiatry.

[bb0670] Wilhelm B., Jordan M., Birbaumer N. (2006). Communication in locked-in syndrome: effects of imagery on salivary pH. Neurology.

[bb0675] Williams A.D., Blackwell S.E., Mackenzie A., Holmes E.A., Andrews G. (2013). Combining imagination and reason in the treatment of depression: A randomized controlled trial of internet-based cognitive bias modification and internet-CBT for depression. Journal of Consulting and Clinical Psychology.

[bb0680] Williams A.D., Moulds M.L. (2007). An investigation of the cognitive and experiential features of intrusive memories in depression. Memory.

[bb0685] Williams A.D., O'Moore K., Blackwell S.E., Smith J., Holmes E.A., Andrews G. (2015). Positive imagery cognitive bias modification (CBM) and Internet-based cognitive behavioural therapy (iCBT): A randomized controlled trial. Journal of Affective Disorders.

[bb0690] Witvliet C.V., Vrana S.R. (1995). Psychophysiological responses as indexes of affective dimensions. Psychophysiology.

[bb0695] Wolpe J. (1958). Psychotherapy by reciprocal inhibition.

[bb0700] Wolpe J. (1973). The practice of behavior therapy.

[bb0705] Zinbarg R.E., Craske M.G., Barlow D.H. (2006). Mastery of your anxiety and worry (MAW): Therapist guide.

